# Hyperoxemia and long-term outcome after traumatic brain injury

**DOI:** 10.1186/cc12856

**Published:** 2013-08-19

**Authors:** Rahul Raj, Stepani Bendel, Matti Reinikainen, Riku Kivisaari, Jari Siironen, Maarit Lång, Markus Skrifvars

**Affiliations:** 1 Department of Neurosurgery, Helsinki University Central Hospital, Topeliuksenkatu 5 FI-00029 HUS Helsinki,Finland; 2 Department of Intensive Care Medicine, Kuopio University Hospital and Kuopio University, Puijonlaaksontie 2, 70211 Kuopio, Finland; 3 Department of Intensive Care Medicine, North Karelia Central Hospital, Tikkamäentie 16, 80210 Joensuu, Finland; 4 Department of Anesthesiology and Intensive Care Medicine, Helsinki University Central Hospital, Helsinki, Finland

**Keywords:** Arterial oxygen tension, Neurocritical care, Oxygenation, Traumatic brain injury, Hyperoxemia, Intensive care, Mortality, Mechanical ventilation

## Abstract

**Introduction:**

The relationship between hyperoxemia and outcome in patients with traumatic brain injury (TBI) is controversial. We sought to investigate the independent relationship between hyperoxemia and long-term mortality in patients with moderate-to-severe traumatic brain injury.

**Methods:**

The Finnish Intensive Care Consortium database was screened for mechanically ventilated patients with a moderate-to-severe TBI. Patients were categorized, according to the highest measured alveolar-arterial O_2_ gradient or the lowest measured PaO_2_ value during the first 24 hours of ICU admission, to hypoxemia (<10.0 kPa), normoxemia (10.0 to 13.3 kPa) and hyperoxemia (>13.3 kPa). We adjusted for markers of illness severity to evaluate the independent relationship between hyperoxemia and 6-month mortality.

**Results:**

A total of 1,116 patients were included in the study, of which 16% (n = 174) were hypoxemic, 51% (n = 567) normoxemic and 33% (n = 375) hyperoxemic. The total 6-month mortality was 39% (n = 435). A significant association between hyperoxemia and a decreased risk of mortality was found in univariate analysis (*P* = 0.012). However, after adjusting for markers of illness severity in a multivariate logistic regression model hyperoxemia showed no independent relationship with 6-month mortality (hyperoxemia vs. normoxemia OR 0.88, 95% CI 0. 63 to 1.22, *P* = 0.43; hyperoxemia vs. hypoxemia OR 0.97, 95% CI 0.63 to 1.50, *P* = 0.90).

**Conclusion:**

Hyperoxemia in the first 24 hours of ICU admission after a moderate-to-severe TBI is not predictive of 6-month mortality.

## Introduction

Traumatic brain injury (TBI) is the leading cause of mortality and morbidity among the young population [1,2]. Hypoxemia has been shown to be detrimental after TBI [3,4]. Accordingly, guidelines from the European Brain Injury Consortium (EBIC) recommend an arterial oxygen tension (PaO_2_) target of 13.3 kPa (100 mmHg) [5]. The Brain Trauma Foundation (BTF) guidelines recommend that PaO_2_ values lower than 8.0 kPa (65 mmHg) should be avoided, but due to lack of strong evidence an upper limit of PaO_2_ has not been established.

Brain hypoxia (low brain tissue oxygen tension, PbtO_2_) is an independent predictor of poor outcome, regardless of intracranial pressure (ICP), cerebral perfusion pressure (CPP) and injury severity [6]. Lately, there has been growing evidence that patient outcome is improved after applying a PbtO_2_-targeted therapy [7,8]. In PbtO_2_-targeted therapy, high inspired oxygen fraction in percent (FiO_2_) is frequently used to maintain adequate PbtO_2_[9,10]. As a consequence of the high FiO_2_, PaO_2_ increases to supra-physiological levels, that is, hyperoxemia [11]. However, the relationship between hyperoxemia and outcome in patients with TBI is controversial [12,13]. Some clinical studies have reported a significant relationship between hyperoxemia and an increased risk of death, whereas some studies have shown no such relationship or even increased survival for TBI patients with mild hyperoxemia [14-16]. Accordingly, we performed a retrospective observational multicenter study using a large national database to determine the independent relationship between hyperoxemia during the first 24 h after ICU admission following TBI, and long-term mortality.

## Materials and methods

### Finnish Intensive Care Consortium database

The Finnish Intensive Care Consortium (FICC) database is a high quality multicenter database consisting of data from ICUs in 22 different hospitals [17]. The FICC was established in 1994 as a cooperative benchmarking project, the goal of which was to improve the quality of intensive care in Finland. Physiological data are stored by clinical information systems that automatically collect data from patient monitors, ventilators and laboratory systems. Data on comorbidities, type of admission, diagnosis, and outcome are entered manually by ICU staff into the electronic database. Patients admitted after TBI are coded as such. Data are then transferred to the central database, which is processed by Tieto Healthcare & Welfare Ltd. (Kuopio, Finland). Before integration to the central database, automatic filters and specially trained personnel validate the data.

### Data collection, extraction and oxygen values

The ethical committee of the Northern Savonia hospital district approved the study in May 2011 and following that the FICC management committee granted us access to the database. Data were extracted for all patients entered into the FICC database between 2003 and 2012, who had had moderate-to-severe TBI (Glasgow coma scale (GCS) score 3 to 12) and had been admitted to a neurosurgical hospital (five out of twenty-two hospitals). Treatment standards in all included hospitals are according to the BTF cerebral perfusion pressure (CPP)/ICP-directed guidelines [6]. Patients who had been re-admitted, were non-mechanically ventilated, or for whom arterial blood gas analysis (ABG) or long-term outcome data were missing were excluded. Only patients between the ages of 14 to 99 years were included to be able to properly compare the study population with the nested cohort.

The FICC database contains only one PaO_2_ value. The value is chosen according to acute physiology and chronic health evaluation (APACHE) II methodology: that is, the PaO_2_ value associated with the ABG (taken during the first 24 h of ICU admission) with the highest alveolar-arterial (A-a) gradient for patients receiving FiO_2_ ≥0.5 or the ABG associated with the lowest PaO_2_ value for patients receiving FiO_2_ <0.5 (PaO_2_). The following variables were extracted from the FICC database: diagnosis, type of admission, year of admission, APACHE II scores [18], therapeutic intervention scoring system 76 (TISS-76) [19], treatment restrictions, comorbidities, diagnosis, physiological parameters, laboratory parameters in the ICU, and in-hospital and 6-month mortality.

### Statistical analysis

For all statistical analyses we used SPSS Statistics for Windows, Version 20.0, released 2011 (IBM Corp, Armonk, NY, USA). The *χ*^2^ test (two-tailed) was used for categorical univariate analysis. Continuous variables were analyzed for skewness and the appropriate statistical test used accordingly. All continuous variables were highly skewed, hence, the non-parametric Mann–Whitney *U*-test was used. Data are presented as median values with IQR unless otherwise mentioned. The Spearman correlation coefficient was used to assess correlation between variables. The variance inflation factor (VIF) was used to control for co-linearity between variables in multivariate analysis.

Patients were divided according to the collected PaO_2_ (highest alveolar-arterial O_2_ gradient or lowest PaO_2_ value). Arterial oxygen tension levels were defined a priori to analysis: hypoxemia was defined as <10.0 kPa, normoxemia as 10.0 to 13.3 kPa and hyperoxemia as >13.3 kPa [20]. The primary outcome was 6-month mortality and the secondary outcome was in-hospital mortality.

As a marker of illness severity the APACHE II score was used. However, since PaO_2_ is included in the APACHE II model (and we sought to investigate the independent relationship between hyperoxemia and mortality) an adjusted APACHE II index of illness severity independent of PaO_2_ was calculated (AP2no-ox). Furthermore, age and the GCS have been shown to be very strong independent predictors of outcome in TBI, so we tested whether the performance of AP2no-ox increased when recalibrating the index using age and the GCS as separate variables [21]. The final AP2no-ox with the best performance contained only the GCS as a separate variable. The performance of the APACHE II score and the calculated APACHE II index (AP2no-ox) to predict outcome was assessed by calculating the area under the curve (AUC) and the Hosmer-Lemeshow goodness-of-fit test (*R*_*L*_^2^).

A set of predefined potential confounding factors was included in a multivariate analysis using logistic regression to investigate the independent effect of hyperoxemia on outcome. The final multivariate model included: AP2no-ox, PaO_2_ groups, year of admission (before or after 2007), emergency operation, ICP monitoring, controlled hypothermia, and platelet count. Furthermore, the predicted probability of mortality was calculated using multivariable analysis. A locally weighted scatterplot smoothing (lowess) curve was used to show the underlying relationship between PaO_2_ and 6-month mortality. The *R*_*L*_^2^ test was used to assess how predicted and observed mortality matched.

### Nested cohort analysis

Because the FICC database only contained one PaO_2_ value, and data regarding TBI severity were limited, we studied an additional nested cohort of TBI patients to better understand the descriptive value of the PaO_2_ measured according to the APACHE II methodology. Furthermore, we wanted to control for co-linearity between TBI severity and PaO_2_. The nested cohort consisted of patients with a moderate-to-severe TBI treated in the ICU of a designated tertiary neurosurgical trauma center (Töölö Hospital, Helsinki University Central Hospital, Finland) between 1 January 2009 and 31 December 2010. Only patients mechanically ventilated during the first 24 h of ICU admission were included. TBI severity was measured using the IMPACT-TBI prognostic model. The International Mission for Prognosis and Clinical Trials (IMPACT)-TBI model predicts risk of 6-month mortality based on patient admission characteristics [21]. All ABG data during the mechanical ventilation period were collected and used to calculate a time-weighted average of PaO_2_ (TWA-O_2_). Furthermore, as in the main study population, the PaO_2_ value associated with the highest A-a gradient or lowest oxygen value was collected (nPaO_2_). To evaluate the descriptive value of PaO_2_ we tested for correlation between TWA-O_2_ and nPaO_2_. To control for the relationship between TBI severity and PaO_2_ correlation between TWA-O_2_, we tested the nPaO_2_ and the IMPACT score. Correlation between variables was tested using the Spearman correlation coefficient.

## Results

**Figure 1:**
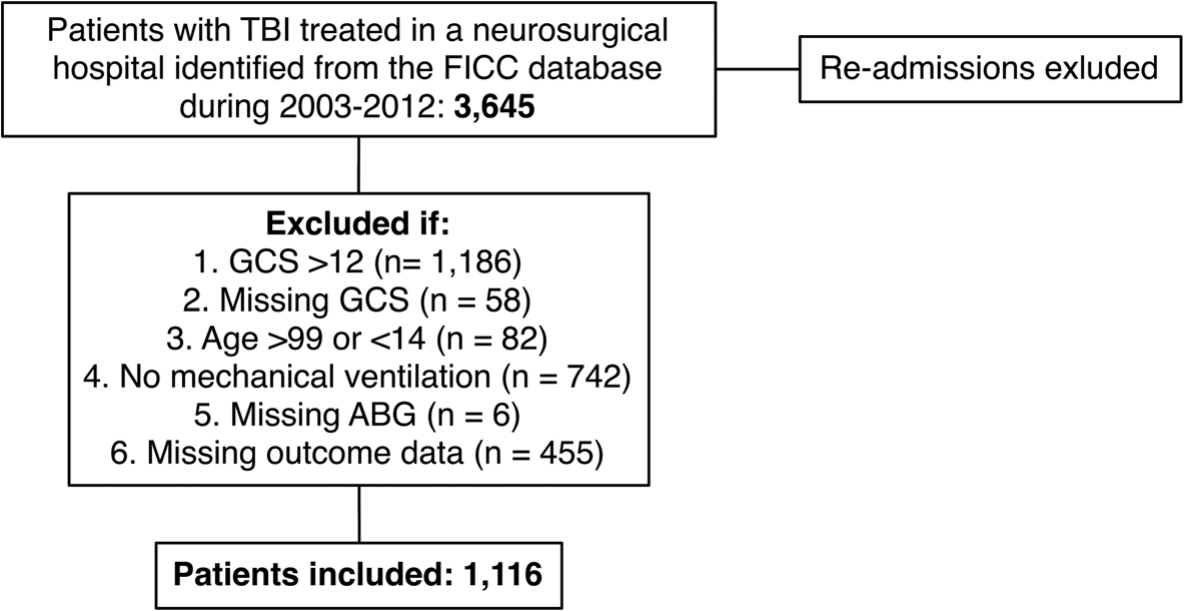
**Study population.** TBI, traumatic brain injury; FICC, Finnish Intensive Care Consortium; GCS, Glasgow coma scale; ABG, arterial blood gas analysis.

A total of 1,116 patients met the inclusion criteria (Figure 1)*.* Of these patients 16% (n = 174) were hypoxemic, 51% (n = 567) normoxemic and 33% (n = 375) hyperoxemic. Baseline characteristics and physiological parameters are presented in Table 1*.* The median age was 53 years (IQR 35 to 64). There were some significant differences between the PaO_2_ groups in baseline characteristics. Hyperoxemic patients were significantly younger than normoxemic and hypoxemic patients (*P* <0.020). Patients in the hypoxemic group had received a significantly higher median FiO_2_ than patients in the normoxemic and hyperoxemic groups (median FiO_2_ 44%, 40%, 39%, *P* <0.001) but had a lower PaO_2_/FiO_2_ ratio than normoxemic and hyperoxemic patients (median PaO_2_/FiO_2_ 19, 31, and 48, respectively; *P* <0.001). Furthermore, it was noted that patients in the hypoxemic group had significantly lower mean arterial pressure (MAP) than normoxemic and hyperoxemic patients (median MAP 69, 105, and 106, respectively; *P* = 0.002).

**Table 1:** Baseline characteristics of the study population

	**All patients (n = 1,116)**	**Hypoxemia (n = 174)**	**Normoxemia (n = 375)**	**Hyperoxemia (n = 567)**	***P*-value**
Age, years	53 (35, 64)	53 (34, 63)	55 (41, 66)	52 (32, 64)	0.020
Year of admission					
2002 to 2007	461 (41)	83 (48)	179 (48)	199 (35)	<0.001
2008 to 2012	655 (59)	19 (52)	196 (52)	368 (65)	
Emergency operation	576 (52)	87 (50)	187 (50)	302 (53)	0.515
Operative admission	632 (57)	82 (47)	218 (58)	332 (59)	0.022
Controlled hypothermia	84 (8)	5 (3)	25 (7)	54 (10)	0.011
ICP monitoring	507 (46)	72 (41)	150 (40)	285 (50)	0.004
Treatment restriction	206 (19)	33 (19)	68 (18)	105 (19)	0.972
Platelets (10^9^)	168 (118, 225)	156 (99, 231)	153 (111, 220)	174 (123, 225)	0.250
**Markers of injury severity**					
APACHE II score	24 (19, 28)	27 (22, 32)	24 (19, 28)	23 (19, 27)	<0.001
Glasgow Coma Scale					
3 to 5	657 (59)	118 (68)	221 (59)	318 (56)	0.047
6 to 8	267 (24)	38 (22)	86 (23)	143 (25)	
9 to 12	192 (17)	18 (10)	68 (18)	106 (19)	
TISS-76 total score	133 (69, 287)	116 (69, 284)	131 (70, 278)	138 (68, 314)	0.803
TISS-76 average score	33 (29, 38)	35 (29, 39)	32 (28, 37)	33 (29, 38)	0.012
ICU length of stay, days	3 (1, 7)	2 (1, 7)	3 (1, 7)	3 (1, 8)	0.309
Hospital length of stay, days	6 (3, 14)	7 (3, 15)	6 (2–11)	7 (3, 14)	0.016

Continuous variables are presented as median (IQR) and categorical variables as n (%); ICP, intracranial pressure; APACHE II, acute illness severity and chronic health evaluation II; TISS-76, therapeutic intervention scoring system 76.

**Table 2:** Unadjusted outcomes

	**All patients (n = 1116)**	**Hypoxemia (n = 174)**	**Normoxemia (n = 375)**	**Hyperoxemia (n = 567)**	***P*-value**
Mortality, number of patients (%)					
In-ICU	201 (18)	42 (24)	61 (16)	98 (17)	0.067
In-hospital	313 (28)	64 (37)	105 (28)	144 (25)	0.014
6-month	435 (39)	83 (48)	151 (40)	201 (35)	0.012

**Table 3:** **Adjusted outcomes by multivariable logistic regression model showing relationship between PaO**_**2 **_**groups and outcome**

**Variable**	**Odds ratio (95% CI)**	***P*-value**
	6-month mortality	
Hypoxemia versus normoxemia	0.90 (0.57, 1.41)	0.648
Hyperoxemia versus normoxemia	0.88 (0.63, 1.22)	0.429
Hyperoxemia versus hypoxemia	0.97 (0.63, 1.50)	0.898
	In-hospital mortality	
Hypoxemia versus normoxemia	1.01 (0.63, 1.62)	0.967
Hyperoxemia versus normoxemia	0.94 (0.65, 1.36)	0.753
Hyperoxemia versus hypoxemia	0.93 (0.59, 1.47)	0.766

Multivariate analysis adjusted for acute physiology and chronic health evaluation II (APACHE II) index independent of oxygenation (AP2no-ox), admission year (before or after 2007), emergency operation, intracranial pressure monitoring, controlled hypothermia and platelet count. PaO_2 _(arterial oxygen tension) groups: hypoxemia, <10.0 kPa; normoxemia, 10.0 to 13.3 kPa; hyperoxemia, >13.3 kPa. The multivariable model displayed excellent discrimination (area under the curve 0.82, 0.83) and calibration (*R*_*L*_^2^ = 0.708, 0, 119) for predicting 6-month mortality and in-hospital mortality, respectively.

Some slight variations in GCS between the groups were noted (*P* = 0.047). Also, controlled hypothermia and ICP monitoring were more frequently done in the hyperoxemia group (*P* = 0.011 and 0.004, respectively). However, there were no significant differences in overall treatment according to the TISS-76 (*P* = 0.803), but hyperoxemic patients had a higher average daily TISS-76 score than normoxemic and hypoxemic patients (*P* = 0.012).

The median APACHE II score for the whole cohort was 24 (IQR 19 to 28). The score was significantly higher in the hypoxemic group compared to the hyperoxemic and normoxemic group (*P* <0.001). Median AP2no-ox was lowest in the hyperoxemia group (28.5, IQR 13.5 to 58.4) followed by the normoxemia group (35.5, IQR 15.1 to 58.4) and highest in the hypoxemia group (49.1, IQR 19.7 to 71.1) (*P* <0.001). The APACHE II score showed excellent performance for predicting 6-month mortality in our patient cohort, with an AUC of 0.80 and an R_L_^2^ of 0.10. The APACHE II index (AP2no-ox) also showed excellent performance for predicting 6-month mortality, with an AUC of 0.82 and an R_L_^2^ of 0.32. Patients excluded due to missing data on long-term outcome did not significantly differ in PaO_2_ (13.1 kPa, IQR 10.8 to 16.8) (*P* = 0.394), but they had a slightly lower APACHE II score (22, IQR 19 to 26) than the included patients (*P* = 0.001).

### Nested cohort

A total of 298 patients were included in the nested cohort (Additional file 1). An average of 6.3 ABG was collected for every patient in the nested cohort. The median TWA-O_2_ was 20.9 kPa (IQR 17.1 to 25.2) and the median nPaO_2_ was 18.4 kPa (IQR 13.7 to 24.9). The median IMPACT score was 38 (IQR 22 to 54). No statistical significant correlation between the IMPACT score and nPaO_2_ or TWA-O_2_ was found (Spearman correlation coefficient −0.027 (*P* = 0.638) and −0.19 (*P* = 0.741), respectively) (Additional file 2). Hence, we showed that there was no co-linearity between TBI severity and PaO_2_**.** Furthermore, there was statistically significant correlation between TWA-O_2_ and nPaO_2_ (Spearman correlation coefficient 0.688, *P* <0.001) (Additional file 3). Thus, we established that the PaO_2_ value chosen using the APACHE II methodology accurately describes the patients’ oxygenation state during the whole mechanical ventilation period and is not influenced by TBI severity.

### Outcome

**Figure 2:**
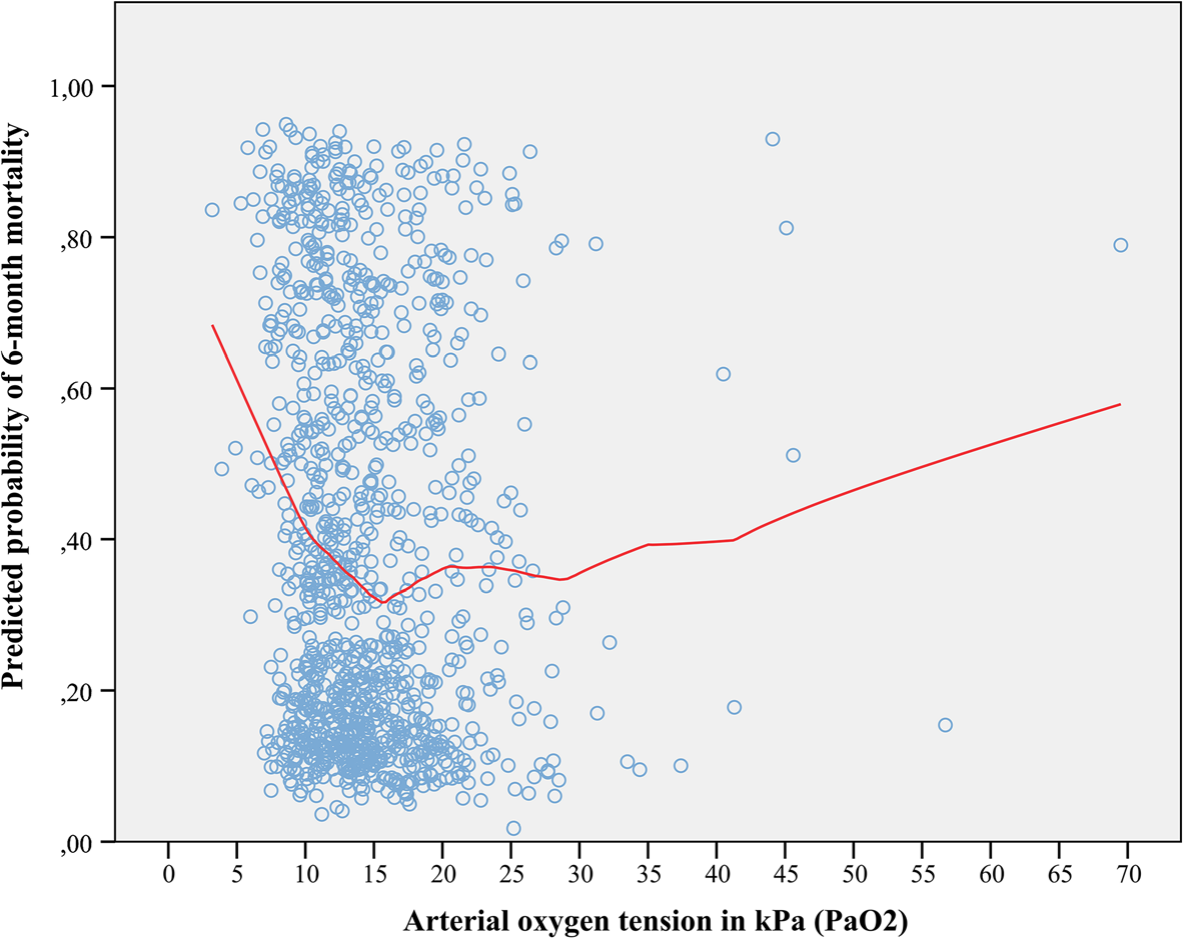
**Locally weighted scatterplot smoothing (lowess) curve showing the relationship between arterial oxygen value (PaO**_**2**_**) and predicted 6-month mortality.** Predicted risk of death showed good performance in predicting actual mortality with an area under the curve (AUC) of 0.869 and *R*_*L*_^2^ of 0.422. A clear association between increased risk of death and low (approximately <11 kPa) PaO_2_ or values very high (approximately >42 kPa) PaO_2_ values was noted. The predicted probability of death is calculated using the following variables: acute physiology and chronic health evaluation II (APACHE II) index independent of oxygenation (AP2no-ox), admission year (before or after 2007), emergency operation, intracranial pressure monitoring, controlled hypothermia and platelet count.

**Figure 3:**
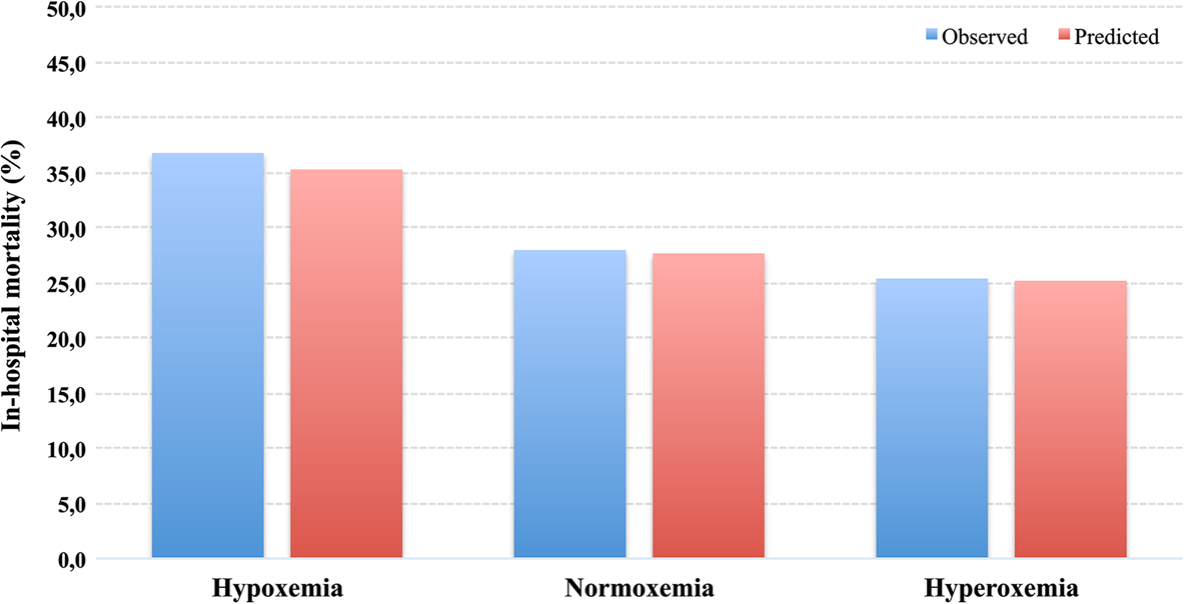
**Observed and mean predicted in-hospital mortality differences between arterial oxygen tension (PaO**_**2**_**) groups.** The difference in mean predicted risk of death was significantly different among the groups (*P* <0.001), it being highest in the hypoxemia group and lowest in the hyperoxemia group. Predicted risk of death matched observed mortality very well within the quartiles with *R*_*L*_^2^ values between 0.097 and 0.746. The predicted probability of death was calculated using the following variables: acute physiology and chronic health evaluation II (APACHE II) index independent of oxygenation (AP2no-ox), admission year (before or after 2007), emergency operation, intracranial pressure monitoring, controlled hypothermia and platelet count.

**Figure 4:**
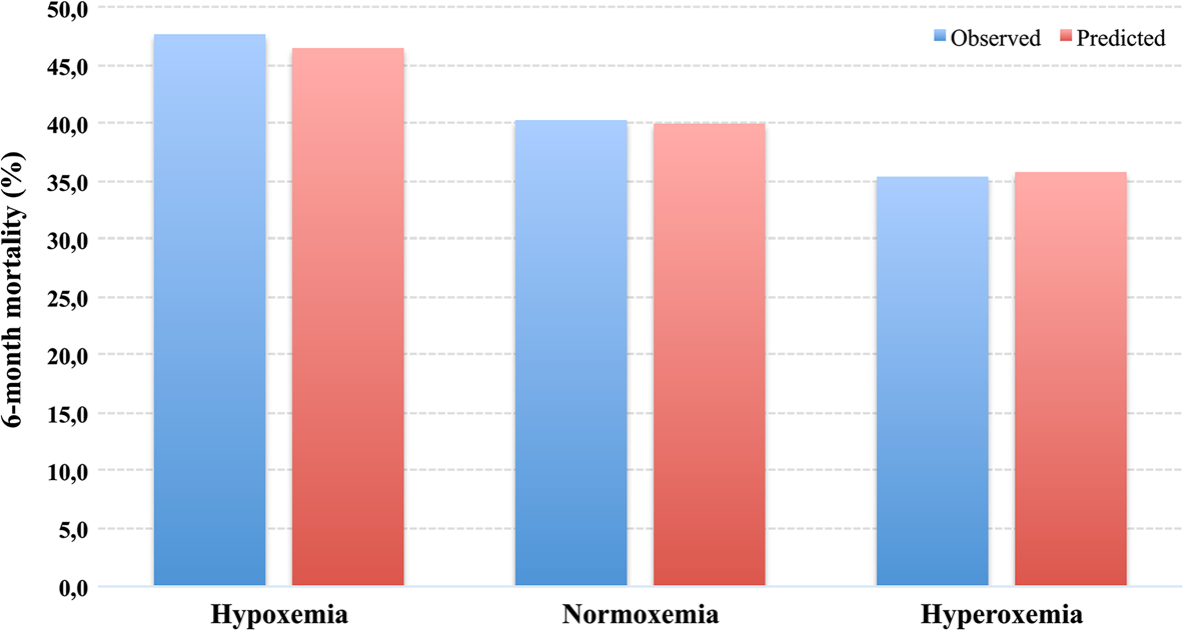
**Observed and mean predicted 6-month mortality differences between PaO**_**2 **_**groups.** The difference in mean predicted risk of death is significantly different among the groups (p < 0.001), being highest in the hypoxemia group and lowest in the hyperoxemia group. Predicted risk of death matched observed mortality very well within the quartiles with R_L_^2^ values between 0.519 and 0.603.

**Figure 5:**
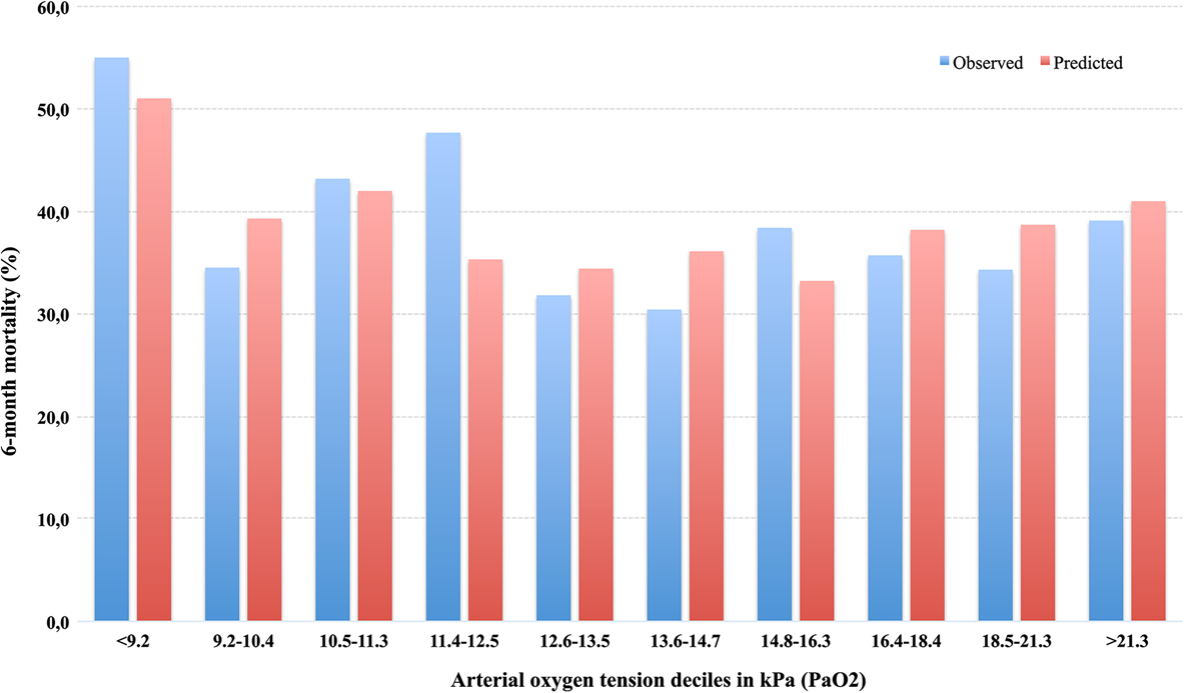
**Observed and predicted 6-month mortality by deciles of arterial oxygen tension (PaO**_**2**_**).** PaO_2_ was divided by deciles. Predicted risk of death matched observed mortality very well within deciles with *R*_*L*_^2^ values between 0.088 and 0.987. The predicted probability of death was calculated using the following variables: acute physiology and chronic health evaluation II (APACHE II) index independent on oxygenation (AP2no-ox), admission year (before or after 2007), emergency operation, intracranial pressure monitoring, controlled hypothermia and platelet count.

Unadjusted outcomes are presented in Table 2*.* The overall total 6-month mortality was 39% (n = 435). Of the non-survivors, 46% (n = 201) died in the ICU and 72% died in hospital before they could be discharged. In univariate analysis, hyperoxemic patients had significantly lower 6-month and in-hospital mortality compared to normoxemic and hypoxemic patients (*P* = 0.012 and 0.014, respectively). However, after adjusting for confounding factors in a multivariate logistic regression model, hyperoxemia had no independent relationship with 6-month mortality compared to normoxemia and hypoxemia: for hyperoxemia versus normoxemia, odds ratio (OR) = 0.88, 95% CI 0.63, 1.22 (*P* = 0.429); for hyperoxemia versus hypoxemia, OR = 0.97, 95% CI 0.63, 1.50 (*P* = 0.898) (Table 3). The same results were noted for in-hospital mortality. There was no significant co-linearity between variables in the final multivariate analysis (VIF_max_ = 1.13). The underlying relationship between predicted risk of death and PaO_2_ is shown with a lowess smoother curve in Figure 2. The mean predicted risk for 6-month mortality was 38.9% (SD 27.0) and for in-hospital mortality it was 27.7% (SD 25.0). There was significant variation between the groups in the predicted probability of death, it being highest in the hypoxemia group and lowest in the hyperoxemia groups (*P* <0.001) (Figures 3 and 4). To further investigate the relationship between hyperoxemia and outcome, PaO_2_ values were divided by deciles. Hyperoxemic PaO_2_ deciles were compared to normoxemic deciles in a multivariate analysis adjusting for same variables as above. However, even after dividing PaO_2_ by deciles, no statistically significant association between hyperoxemia and outcome was noted (Figures 5).

## Discussion

### Key findings

We conducted a large multicenter retrospective observational study investigating the relationship between hyperoxemia in the first 24 h after ICU admission and long-term mortality in patients with moderate-to-severe TBI. Initially, in univariate analysis a significant association between hyperoxemia and decreased risk of death was noted. However, after adjusting for illness severity in multivariate analysis, no association between hyperoxemia and outcome was noted. The results remained when dividing PaO_2_ by deciles. Thus, no consistently reproducible independent relationship between hyperoxemia and outcome was determined.

### Comparison with other studies

The deleterious effect of hypoxemia in TBI patients is well known [3]. Acknowledge guidelines advocate PaO_2_ values between 8.0 and 13.3 kPa (60-100 mmHg) [5,6,20].

Normobaric hyperoxia therapy during ICU care is a commonly used treatment alternative providing a safe margin to hypoxemia [22,23]. However, the use of hyperoxia (normobaric) is not without problems and experimental research has provided data indicating harmful effects of hyperoxia exposure due to increased free radical damage, activation of programmed cell death pathways and expression of pro-inflammatory and anti-inflammatory cytokines, ultimately leading to cell death and causing acute lung injury, with similar histopathological findings to acute respiratory distress syndrome (ARDS) [24-26]. Previous studies have shown that approximately 20 to 30% of all patients with severe TBI develop acute lung injury (ALI)/ARDS, resulting in poorer long-term outcomes [27]. Suggested etiologies of ALI/ARDS in patients with TBI include the use of high tidal volume, high respiratory rate, aspiration, pneumonia, and neurogenic pulmonary edema [28]. Furthermore, normobaric hyperoxia therapy has been shown to cause cerebral vasoconstriction, reducing cerebral perfusion, which may potentially increase cerebral ischemia [29,30]. However, experimental studies by Singhal *et al*. have suggested that the benefits of normobaric hyperoxia exposure outweigh the risks [31]. The exact mechanism of hyperoxia-induced lung injury remains incompletely understood and there is not enough evidence from clinical studies showing that normobaric hyperoxia treatment (that is, high FiO_2_) increases the risk of ALI independent of the underlying disease (for example, bacterial or viral infections, trauma, chronic lung injury, aspiration, or lung contusion) [25]. Despite the potentially harmful effects, normobaric hyperoxic therapy has been shown to be beneficial in treating low brain oxygen levels, together with ICP control and CPP maintenance [11,32,33]. When normobaric hyperoxia is applied it is aimed at keeping PbtO2 greater than 20 to 25 mmHg (2.7 to 3.3 kPa), which is 50% of the normal brain tissue oxygen levels [34]. Also, the increased oxygen availability with high FiO_2_ levels may lead to induction of cerebral aerobic metabolism, alleviating ischemic injury [35,36].

In 2007 the BTF guidelines presented class III evidence in favor of a PbtO_2_-targeted therapy in combination with the traditional CPP/ICP-targeted therapy [7]. Newer studies have reinforced this by showing improved patient outcomes when using the combined PbtO_2_ therapy compared to using the traditional CPP/ICP therapy [37,38]. In a review article by Nangunoori *et al*. the use of PbtO_2_-targeted therapy doubled the likelihood of a favorable neurological outcome in patients with TBI [8]. There are several treatment strategies for maintaining PbtO_2_, increasing FiO_2_ and augmenting CPP by ICP-lowering treatment, vasopressors and fluids being the most commonly used [9,39]. However, in the presence of cerebral ischemia, aggressive attempts to maintain CPP with fluids and vasopressors should be avoided due to risk of ALI/ARDS [6,40]. Thus, increasing FiO_2_ to achieve supra-physiological arterial oxygen tension levels (hyperoxemia) has a central role in treating a lowered PbtO_2_, especially in the presence of adequate ICP and CPP [37,41]. Whether increasing oxygen in arterial blood to supra-physiological levels has a positive impact on cerebral metabolism and improves outcomes remains to be debated [10,42].

Clinical studies investigating the relationship between hyperoxia therapy or hyperoxemia and outcome in TBI patients have come up with controversial results. Tolias *et al*. showed in a prospective non-randomized study that hyperoxia therapy significantly improved cerebral oxidative metabolism and decreased ICP. This was associated with better outcomes, supporting the use of hyperoxia therapy [32]. Davis *et a*l. showed a reduced risk of in-hospital mortality in patients with mild hyperoxemia (PaO_2_ 15 to 65 kPa) on admission, but increased risk of death in patients with extreme hyperoxemia (>65 kPa). In a small, retrospective, single-center study including 193 severe TBI patients, Asher *et al*. showed improved survival for hyperoxemic patients whose PaO_2_ thresholds were between 33 and 65 kPa during the first 72 h of admission [43]. In a large, single-center, retrospective study including 1,547 patients, Brenner *et al*. showed a significant association between hyperoxemia (PaO_2_ >26.6 kPa) and poor short-term outcome after TBI. However, the study was not restricted to patients on mechanical ventilation, which may induce bias, because it is probable that patients not on mechanical ventilation are likely to have a less severe TBI and hyperoxemia is more likely with mechanical ventilation [44]. In this study we avoided this potential confounding factor by excluding all patients not on mechanical ventilation. Similar to the present study, Eastwood *et al*. found no association between early hyperoxemia in general ICU patients during the first hours of ICU stay, and in-hospital mortality [45]. This was further confirmed by Young *et al*. studying mechanically ventilated ischemic stroke [46]. Furthermore, studies investigating the relationship between hyperoxemia and outcome in cardiac arrest patients have also yielded controversial results [47,48].

In the present study 6-month mortality was 39%, which is somewhat higher than previously described. The IMPACT study showed a 6-month mortality rate of 32%, and the Corticosteroid Randomization After Significant Head Injury (CRASH) study showed a 29% death rate [21,49]. However, one major factor that has to be considered is the difference in the age of the study populations, as age is a major prognostic factor in TBI patients [50]. The median age in our study was 53 years (IQR 35 to 64) compared to 30 years (IQR 21 to 45) and 32 years (IQR 28 to 47) in the IMPACT and CRASH studies, respectively [21,49].

In this study we did not find any statistically significant reproducible association between hyperoxemia and 6-month mortality in TBI patients treated in the ICU. This could be a consequence of lack of power. However, our study suggests that hyperoxemia is safe and a viable therapy target when trying to avoid the detrimental effects of hypoxemia and subsequent brain hypoxia. Our analysis should be extended in future studies to include all oxygenation values during the first days of TBI treatment. Furthermore, further studies should aim to assess PbtO_2_ levels, as this has shown to improve outcome [37,38,41]. Currently, two highly anticipated studies are underway investigating the role of normobaric hyperoxia therapy (BRAINOXY) and the role of PbtO_2_ targeted therapy (BOOST 2) in patients with TBI in the ICU.

### Strengths and limitations

Our study has several strengths. First, it includes 1,116 patients, making it one of the largest studies of its type conducted so far. Second, our data come from a large, multicenter high-quality database reflecting almost all ICUs in Finland [17]. Third, this is the first study of its kind using 6-month mortality as primary endpoint, which considerably strengthens the credibility of this study, as it has been shown that in-hospital mortality severely underestimates mortality in TBI patients [49]. We acknowledge some limitations with our study. Most importantly the only available oxygen value was the worst one, according to the APACHE II methodology, which carries the risk of not fully describing the patient’s oxygenation situation during the whole mechanical ventilation period. However, as we showed with the nested cohort analysis the PaO_2_ measured using the APACHE II methodology describes the mechanical ventilation period very well. Also, our statistical approach is partly limited to standard statistical methods comparing three levels of oxygenation, which, however, is a physiological process and not linear in nature. However, the lowess smoother analysis (Figure 2) does not assume any linearity in association. Second, we cannot control for oxygen exposure prior to ICU admission. Third, due to the retrospective nature of this study we used the APACHE II model to adjust for illness severity. However, this potential confounding factor was controlled for by performing the nested cohort analysis. Fourth, due to the retrospective nature of the study we were unable to assess long-term neurological outcome and were limited to using long-term mortality as the primary endpoint. Finally, because all institutions use the traditional CPP/ICP-directed therapy as standard TBI care we cannot comment on whether a PbtO_2_-targeted therapy would have improved the outcomes in our study.

## Conclusion

Hyperoxemia in the first 24 h of ICU admission after a moderate-to-severe TBI was not independently associated with 6-month mortality.

## Key messages

•No consistent reproducible relationship between hyperoxemia and risk of death was established.

•Targeting hyperoxemia seems to be a safe approach when trying to maintain adequate brain oxygenation.

•Extreme hyperoxemia should be used with caution.

## Abbreviations

A-a gradient: Alveolar-arterial gradient; ABG: Arterial blood gas; ALI: Acute lung injury; AP2no-ox: Adjusted acute physiology and chronic health evaluation 2 independent of arterial oxygen tension; APACHE II: Acute physiology and chronic health evaluation II; ARDS: Acute respiratory distress syndrome; AUC: Area under the curve; BTF: Brain Trauma Foundation; CPP: Cerebral perfusion pressure; EBIC: European Brain Injury Consortium; FICC: Finnish Intensive Care Consortium; FiO2: Inspired oxygen fraction in percent; GCS: Glasgow coma scale; ICP: Intracranial pressure; IMPACT: International Mission for Prognosis and Clinical Trials in traumatic brain injury; kPa: Kilo Pascal; Lowess: Locally weighted scatterplot smoothing; MAP: Mean arterial pressure; nPaO2: Arterial oxygen tension when the alveolar-arterial gradient is the highest or oxygen value the lowest for patients in the nested cohort; OR: Odds ratio; PaO2: Arterial oxygen tension; PbtO2: Brain tissue oxygen tension; TBI: Traumatic brain injury; TISS-76: Therapeutic intervention scoring system 76; TWA-O2: Time-weighted average of arterial oxygen tension during mechanical ventilation; VIF: Variance inflation factor

## Competing interests

The study was funded by a Helsinki University Hospital EVO grant (TYH2012142) and Medicinska Understödsföreningen Liv och Hälsa.

## Authors’ contributions

RR, SB, MR, and MS designed the study. RR drafted the manuscript assisted by MS, SB, JS, and MR. RR, SB, MR, ML, and MS performed the data collection. RR is responsible for integrity of the collected data. The statistical analysis of the data was performed and interpreted by RR, SB, MR, and MS. RR, RK, JS, and MS performed the nested cohort data collection and analysis. All authors contributed to the interpretation of the data and writing of the manuscript. All authors revised the manuscript and approved it in the final form.

## Supplementary Material

Additional file 1: Table S1 Nested cohort analysis patient characteristics. Click here for file

Additional file 2: Figure S1 Correlation between International Mission for Prognosis and Clinical Trials (IMPACT) score in traumatic brain injury (predicted risk for mortality) and arterial oxygen tension when the alveolar-arterial gradient is the highest or oxygen value the lowest (nPaO_2_), measured using the acute physiology and chronic health evaluation II (APACHE II) methodology in patients in the nested cohort. Click here for file

Additional file 3: Figure S2 Correlation between the time weighted average of arterial oxygen tension during the whole mechanical ventilation period (TWA-O_2_) and arterial oxygen tension when the alveolar-arterial gradient is the highest or oxygen value the lowest for patients in the nested cohort (nPaO_2_), measured using the acute physiology and chronic health evaluation II (APACHE II) methodology in patients in the nested cohort. Click here for file
