# Supplementary material for: Hyperoxemia and long-term outcome after traumatic brain injury
Source: Crit Care. 2013 Aug 19;17(4):R177. doi: 10.1186/cc12856 (PMC4056982; doi:10.1186/cc12856)
Supplement: Additional file 1: Table S1 — Nested cohort analysis patient characteristics. [file cc12856-S1.docx]

| **Appendix Table 1: Nested cohort** | | | | |
| --- | --- | --- | --- | --- |
| **Variable** | **Hyperoxemia**  **(> 26.6 kPa)** | **Normoxemia**  **(13.3 – 26.6 kPa)** | **Hypoxemia**  **(< 13.3 kPa)** | **p-Value** |
| **Number of patients** | 59 | 172 | 12 |  |
| **nPaO_2_** (kPa) | 30.7 (28.2 – 35.0) | 18.5 (16.0 – 22.4) | 11.5 (10.1 – 12.6) | < 0.001 |
| **nPaCO_2_** (kPa) | 4.7 (4.0 - 5.2) | 5.0 (4.4 - 5.4) | 5.0 (4.6 - 5.4) | 0.048 |
| **TWA-O_2_** (kPa) | 27.5 (23.4 – 30.6) | 21.0 (18.1 – 23.9) | 16.0 (14.1 – 18.4) | < 0.001 |
| **TWA-CO_2_** (kPa) | 4.6 (4.4 - 5.0) | 4.9 (4.5 - 5.2) | 4.9 (4.5 - 5.3) | 0.015 |
| **TWA-FiO_2_** (%) | 43 (40 – 48) | 42 (38 – 47) | 45 (39 – 53) | 0.023 |
| **IMPACT score** (%) | 39 (19 – 51) | 39 (23 – 55) | 38 (23 – 52) | 0.846 |
| **Age (14-99)** | 48 (26 – 64) | 59 (45 – 67) | 56 (36 – 64) | 0.011 |
| **Motor score ≤ 5** | 54 (92) | 155 (90) | 61 (90) | 0.941 |
| **Pupils** |  |  |  |  |
| **Abnormal** | 30 (51) | 61 (35) | 24 (46) | 0.133 |
| **Normal** | 29 (49) | 111 (65) | 36 (54) |  |
| **Hypoxia*** | 15 (25) | 31 (18) | 20 (30) | 0.112 |
| **Hypotension*** | 7 (12) | 20 (12) | 9 (13) | 0.927 |
| **Marshall CT-classification** |  |  |  |  |
| DI I | 1 (2) | 1 (1) | 2 (3) | 0.259 |
| DI II | 16 (27) | 42 (24) | 9 (13) |  |
| DI III | 0 (0) | 4 (2) | 4 (6) |  |
| DI IV | 0 (0) | 1 (1) | 1 (2) |  |
| EML | 30 (51) | 84 (49) | 39 (58) |  |
| NEML | 12 (20) | 40 (23) | 12 (18) |  |
| **Subarachnoid hemorrhage** | 38 (64) | 119 (69) | 45 (67) | 0.789 |
| **Epidural hemorrhage** | 7 (12) | 8 (5) | 5 (8) | 0.155 |
| **Basal cisterns open** | 40 (68) | 124 (72) | 47 (70) | 0.815 |
| **Midline shift** | 38 (64) | 114 (66) | 48 (72) | 0.646 |
| **In-hospital mortality** | 11 (19) | 32 (19) | 9 (13) | 0.616 |
| **6-month mortality** | 17 (29) | 59 (34) | 21 (31) | 0.719 |
| Categorical variables presented as n (%), continuous variables presented at median (IQR), nPaO_2_ = oxygen value measured using the APACHE 2 methodology, nPaCO_2_ = carbon dioxide values associated with the ABG containing the nPaO_2_ value, IMPACT= international mission for prognosis and analysis of clinical trials in TBI, TBI= traumatic brain injury, TWA= time weighted average, TWA-FiO_2_ = time weighted average inspired oxygen fraction during the whole mechanical ventilation period, CT= computerized tomography, DI= diffuse injury, EML= evacuated mass lesion, NEML= non-evacuated mass lesion, * hypoxia is defined as an oxygen saturation < 90 % and hypotension as a systolic blood pressure below 90 mmHg at any time in the pre-hospital setting. | | | | |
