# Supplementary figures and images for: Hyperoxemia and long-term outcome after traumatic brain injury
Source: Crit Care. 2013 Aug 19;17(4):R177. doi: 10.1186/cc12856 (PMC4056982; doi:10.1186/cc12856)

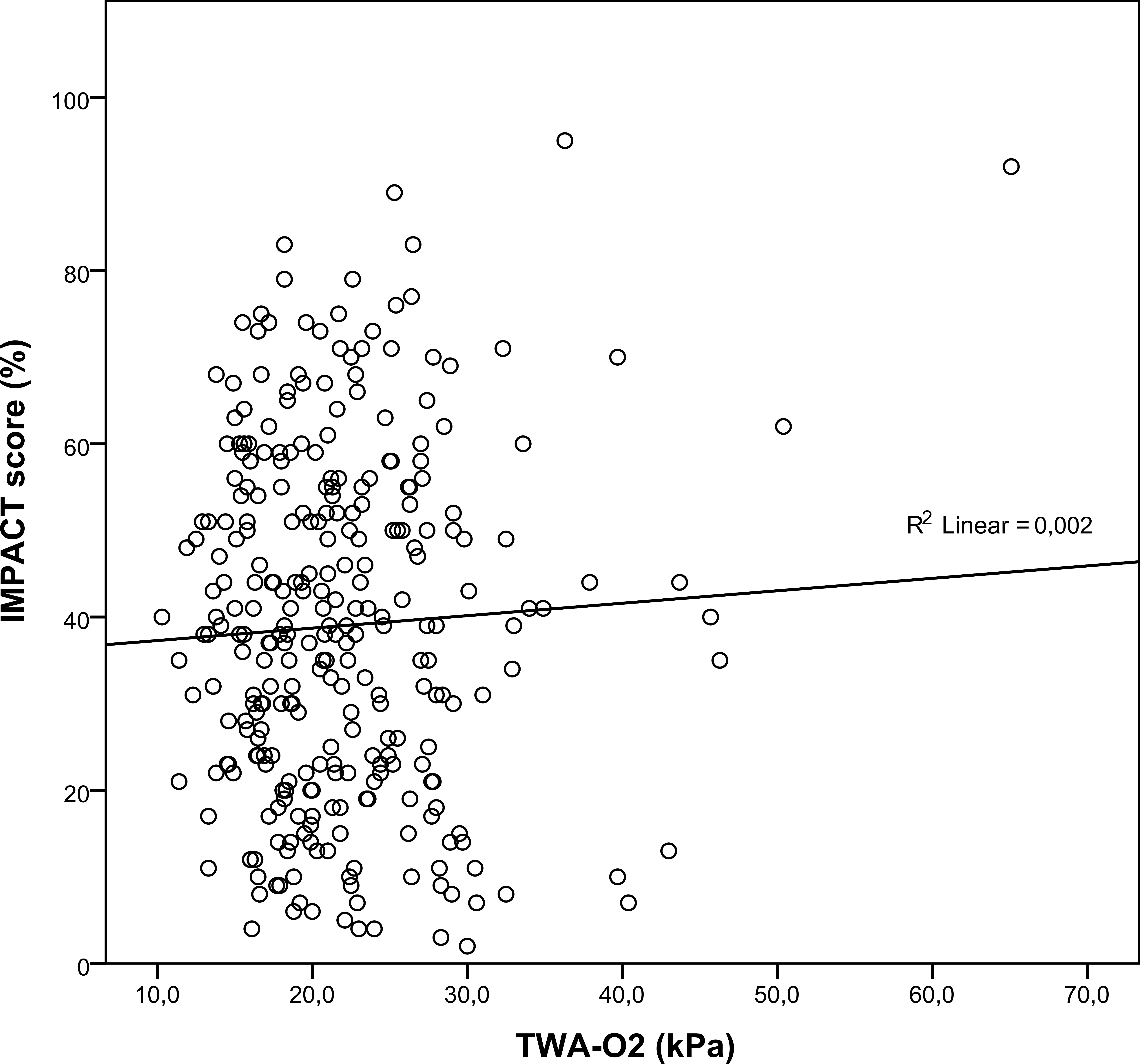

Supplement: Additional file 2: Figure S1 — Correlation between International Mission for Prognosis and Clinical Trials (IMPACT) score in traumatic brain injury (predicted risk for mortality) and arterial oxygen tension when the alveolar-arterial gradient is the highest or oxygen value the lowest (nPaO2), measured using the acute physiology and chronic health evaluation II (APACHE II) methodology in patients in the nested cohort. [file cc12856-S2.tiff]

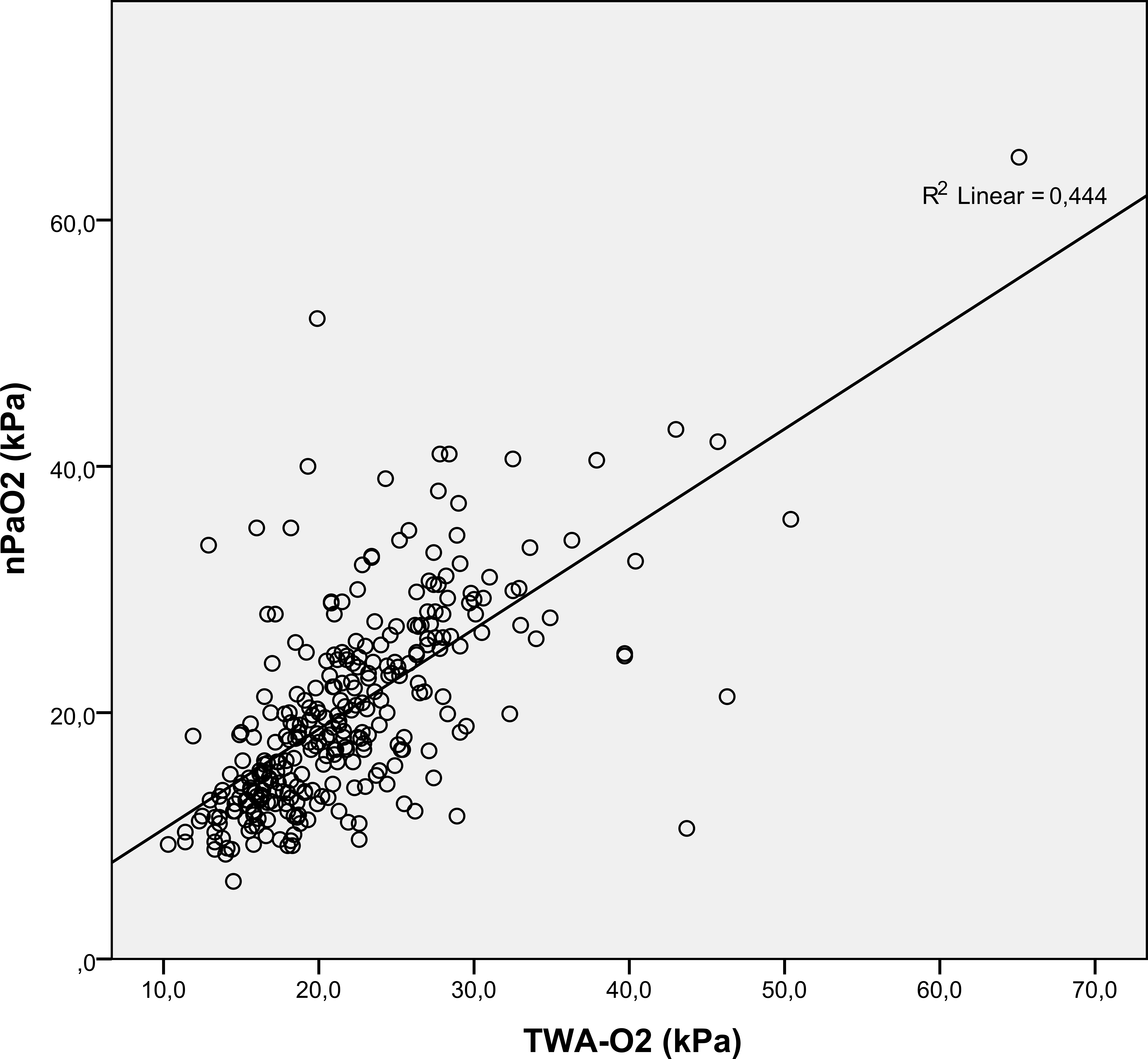

Supplement: Additional file 3: Figure S2 — Correlation between the time weighted average of arterial oxygen tension during the whole mechanical ventilation period (TWA-O2) and arterial oxygen tension when the alveolar-arterial gradient is the highest or oxygen value the lowest for patients in the nested cohort (nPaO2), measured using the acute physiology and chronic health evaluation II (APACHE II) methodology in patients in the nested cohort. [file cc12856-S3.tiff]
